# Detecting continuous and discrete frequency changes as a function of spectral resolvability and modulation rate

**DOI:** 10.1121/10.0044323

**Published:** 2026-07-01

**Authors:** Penelope J. Corbett, Kelly L. Whiteford, Andrew J. Oxenham

**Affiliations:** 1 Department of Psychology, University of Minnesota, Minneapolis, Minnesota 55455, USA; 2 Department of Otolaryngology-Head and Neck Surgery, Kresge Hearing Research Institute, University of Michigan, Ann Arbor, Michigan 4605, USA

## Abstract

This study measured detection of frequency modulation (FM) and frequency differences in alternating tones with spectrally resolved and unresolved harmonics for modulation rates between 2 and 20 Hz. The hypotheses were that FM detection involves (i) conversion to amplitude modulation through cochlear filtering and (ii) sampling instantaneous frequency at discrete times. Contrary to the first hypothesis, thresholds worsened with increasing modulation rate similarly for both resolved and unresolved harmonics. Thresholds for alternating tones were higher than for FM, particularly at faster modulation rates. Outcomes can be reconciled with existing theories if performance is limited by central, not peripheral, processes.

## 1. Introduction

Frequency modulation (FM) provides critical cues for speech, music, and other sounds. Despite its importance, questions remain regarding how it is encoded in the auditory system. The dual-code theory (Moore and Sęk, 1995) posits two mechanisms for detecting FM in pure tones or complex tones with spectrally resolved harmonics: (1) At low carrier frequencies (≲4 kHz) and slow FM rates (≲5 Hz), precise neural phase-locking to the tones& instantaneous frequencies may be used to encode FM. (2) At faster FM rates (>5–10 Hz), the mechanism for tracking the timing of phase-locked neural responses may be too “sluggish” to follow the rapid changes in instantaneous frequency, leading to a decrease in the use of this cue. Similarly, at high carrier frequencies (>4–5 kHz), auditory nerve fibers no longer exhibit strong phase locking to the stimulus carrier, and so phased-locked timing cues may not be available at any FM rate (Sęk and Moore, 1995; Verschooten *et al.*, 2019). In these cases, FM detection may instead be achieved via amplitude-modulation (AM) cues, produced by the FM-to-AM conversion that occurs because of cochlear filtering (Moore and Sęk, 1995; Saberi and Hafter, 1995; Sęk and Moore, 1995; Whiteford *et al.*, 2017; Zwicker, 1956).

One way to test this hypothesis is to compare FM detection thresholds as a function of modulation rate using carriers that include either spectrally resolved harmonics or only unresolved harmonics. In the case of unresolved harmonics, temporal cues are available via the temporal envelope of the stimulus (Houtsma and Smurzynski, 1990; Shackleton and Carlyon, 1994), but FM-to-AM cues should not be available because none of the harmonics are spectrally resolved and so do not create the spectral peaks needed to generate AM as their frequency varies. We might therefore expect FM detection thresholds to worsen more rapidly for unresolved harmonics than for resolved harmonics as the modulation rate increases because listeners cannot transition to using the FM-to-AM code when only unresolved harmonics are present. No clear evidence for or against this prediction is currently available. Although Carlyon *et al.* (2000) found some differences in line with the prediction between 1 and 2 Hz modulation rate, thresholds for rates between 2 and 20 Hz seemed to increase in a similar manner for both resolved and unresolved harmonics. However, their use of only four participants limited the statistical power with which an interaction between stimulus carrier (resolved vs unresolved harmonics) and modulation rate could be detected.

Another question relates to how FM is detected—whether it is via the detection of changes in dynamic portions of the stimulus (Lyzenga *et al.*, 2004) or via the maximum/minimum excursions in frequency, using “snapshots” of the stimulus instantaneous frequency (Demany *et al.*, 2009; Gockel *et al.*, 2001). One way to test these alternatives is to compare patterns of thresholds obtained using continuous FM with thresholds obtained using a steady-state equivalent, where alternating discrete tones are presented to represent the maximum and minimum frequency excursions of FM. Plack and Carlyon (1995) compared continuous FM detection thresholds with discrete tones, using a same-different (SD) task. In the SD task, two tones were presented in each interval of a two-alternative forced-choice (AFC) task, each with a duration equivalent to 0.25 cycles of the 5 Hz FM rate (50 ms) and separated from each other by the same duration (50 ms). In one interval, the tones were presented at the same frequency; in the other interval, the frequency difference between the tones corresponded to the peak-to-peak frequency difference of the equivalent FM condition. This manipulation can be considered as simulating a snapshot of the maximum frequency difference within a single FM cycle. They found that the SD condition produced similar thresholds to single-cycle FM for unresolved harmonics. However, the SD condition produced slightly poorer thresholds than FM for spectrally resolved harmonics for reasons that remained unclear. Other open questions include whether the same pattern of results is observed at modulation rates other than 5 Hz and what thresholds are obtained when the stimuli include more than a single cycle of modulation.

The present study addressed these questions by measuring thresholds for both continuous FM and discrete SD stimuli as a function of modulation rate from 2 to 20 Hz, using an interval duration of 1 s, meaning that at least 2 cycles of modulation were always presented. Based on the FM-to-AM and snapshot hypotheses presented above, our predictions were that (i) FM thresholds should worsen more rapidly as a function of modulation rate with only unresolved harmonics than with resolved harmonics because of the lack of FM-to-AM cues in conditions with only spectrally unresolved harmonics and (ii) FM and SD conditions should produce similar thresholds and similar dependence on modulation rate if the SD condition provides a reasonable approximation of the information provided by FM (as predicted by the snapshot hypothesis).

## 2. Method

### 2.1 Participants

Twenty participants (6 male, 14 female) completed the study, with ages ranging from 19 to 55 years (mean = 24.4, standard deviation = 8.0). All participants had normal hearing, as defined by audiometric thresholds at octave frequencies between 250 and 8000 Hz of 20 dB HL or better in both ears. All participants provided informed consent and received monetary compensation for their time. Experimental protocols were approved by the Institutional Review Board of the University of Minnesota. Three additional participants completed the study but were excluded from the final analysis—two because of high (>6% or 1 semitone) thresholds in the slowest-rate SD conditions, suggesting possible amusia (Peretz *et al.*, 2003), and one because of inconsistent responding, leading to more than 50% of runs failing to converge on thresholds below 100% of the carrier frequency.

### 2.2 FM tones

Reference and target stimuli (*F*0 = 200 Hz) were bandpass filtered with an 8th-order Butterworth filter into one of two passband conditions of 500–1700 Hz or 2400–4000 Hz, including some resolved and only unresolved harmonics of 200 Hz in the passband, respectively (Houtsma and Smurzynski, 1990). The stimuli were presented at 45 dB sound pressure level (SPL) per component in the passband. All stimuli were 1 s in duration, including 12.5-ms raised-cosine onset and offset ramps, and the two intervals in each trial were separated by 500 ms. Target stimuli were sinusoidally modulated at one of four rates (2, 5, 10, or 20 Hz), with the modulator starting phase randomized so that the instantaneous F0 always began at the carrier frequency and either increased or decreased in frequency excursion. Reference stimuli were unmodulated. All components in a complex were generated with the same carrier phase in each trial to produce a maximally “peaky” temporal envelope.

### 2.3 SD tones

The SD stimuli had similar parameters to those of the FM stimuli. All components in the carrier again had the same starting phase to produce a maximally peaky stimulus temporal envelope. Instead of one continuous modulated tone, two unmodulated tone bursts were temporally centered on the frequency extremes of each modulation cycle. Each tone burst had raised-cosine onset and offset ramp durations of 12.5 ms. The half-amplitude duration of each tone burst (and the half-amplitude gap between successive tones) corresponded to a quarter of a period so that they were 125, 50, 25, and 12.5 ms for the 2, 5, 10, and 20 Hz conditions, respectively. The total duration of tone bursts and gaps within an interval summed to 1 s. Target stimuli had alternating tone bursts with lower and higher F0s, with the starting F0 (low vs high) randomized on a trial-by-trial basis. Reference stimuli were repetitions of the same stimulus at the nominal F0 of the carrier.

### 2.4 Bandstop noise

Both FM and SD tones were presented in bandstop-filtered threshold-equalizing noise (TEN; Moore *et al.*, 2000) at 40 dB SPL per equivalent rectangular bandwidth around 1 kHz before filtering to limit the audibility of any distortion products. The noise was created with an 8th-order Butterworth filter with inner cutoff frequencies matching the passband of the target stimulus, either 500–1700 Hz for resolved harmonics or 2400–4000 Hz for unresolved harmonics. The outer cutoff frequencies were 20 and 20 000 Hz. The noise began 300 ms before the onset of the first stimulus within a trial and ended 200 ms after the offset of the last stimulus. The noise was gated on and off with 50-ms raised-cosine ramps.

### 2.5 Sound presentation and calibration

All experiments were created within the AFC software package (Ewert, 2013) in matlab 2023. Stimuli were generated digitally and converted to analog at a sampling rate of 48 kHz using a Lynx E22 sound card (Lynx Studio Technology, Costa Mesa, CA), with 24-bit resolution. The stimuli were presented diotically via open-ear headphones (HD 650; Sennheiser Electronic Corporation, Wedemark, Germany) in a sound-attenuating booth.

### 2.6 Procedure

Participants completed two sessions of the experiment on separate days, with each session lasting up to 2 h. Listeners were divided into two equal groups, with one group completing the FM task first and the other group completing the SD task first. Within each of the two tasks, all conditions were tested once before any were repeated. The order of presentation of spectral condition (resolved or unresolved) was selected randomly for each participant and each repetition. Within each repetition of each spectral condition, the presentation order of the modulation rates was also selected randomly. All tasks used a two-interval, two-AFC paradigm, in which participants were asked to select the interval that contained the modulated sound (FM task) or group of tones with varying F0 (SD task). The target was randomly presented in either the first or second interval with equal *a priori* probability. Virtual buttons on the computer screen (labeled 1 and 2) marked the presentation of each stimulus interval, and participants received visual feedback (“Correct” or “Incorrect”) after each trial. A three-down, one-up adaptive procedure was used to track the 79.4% correct point of the psychometric function (Levitt, 1971).

Each run began with a peak-to-peak frequency excursion (2Δ*f*, where Δ*f* is the frequency excursion from the carrier frequency of each component) in the FM task or a frequency difference of each component in the SD task of 20% of the carrier frequency. The frequency excursion or difference initially varied according to the rules of the adaptive procedure by a factor of 2. After the first upper reversal of the adaptive procedure, the step size was reduced to a factor of 1.41. After two more reversals, the step size was reduced further to a factor of 1.19 for the remaining six reversal points. The FM difference limen (FMDL) or F0 difference limen (F0DL) per adaptive run was calculated as the geometric mean of the peak-to-peak frequency excursions [2*Δf*(%)] or frequency differences at the final six reversal points. Participants completed three runs per stimulus condition. The first run in each condition was treated as practice, and the geometric mean threshold from the remaining two runs of each condition was used to calculate an individual's threshold.

## 3. Results

**Fig. 1.**
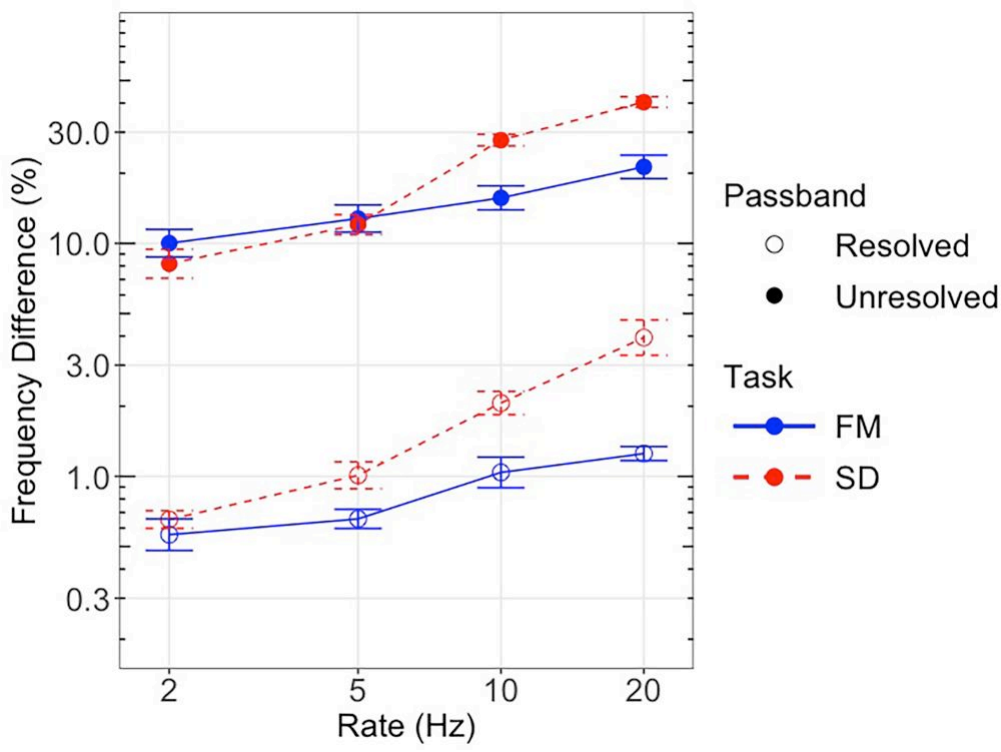
Mean thresholds (*N* = 20) for the two tasks (FM, blue symbols with solid lines; and SD, red symbols with dashed lines) are plotted as peak-to-peak frequency change as a function of modulation rate. Error bars represent ±1 standard error of the mean. The passband condition with resolved harmonics is represented with open symbols, whereas filled symbols represent the passband condition with unresolved harmonics.

The mean results are shown in Fig. 1. A repeated-measures analysis of variance (ANOVA) was conducted on the log-transformed values of FMDLs and F0DLs, with three within-subjects factors of task (FM or SD), modulation rate (2, 5, 10, or 20 Hz), and passband (resolved: 500–1700 Hz vs unresolved: 2400–4000 Hz), and a between-subjects factor of presentation order (FM or SD tested first). Effect sizes were determined using partial-eta-squared ( $\eta_{p}^{2}$*)* for the ANOVA outcomes. Greenhouse–Geisser correction was applied where Mauchly's test indicated a violation of the assumption of sphericity. There was a significant main effect of passband [*F*(1, 18) = 2281, *p* < 0.001, $\eta_{p}^{2}$ = 0.992], modulation rate [*F*(1.83, 32.9) = 189.4, *p* < 0.001, $\eta_{p}^{2}$ = 0.913], and task [*F*(1, 18) = 31.5, *p* < 0.001, $\eta_{p}^{2}$ = 0.637]. The main effects confirmed the effects visible in Fig. 1: thresholds for conditions with unresolved harmonics were consistently higher (poorer) than those with resolved harmonics by about an order of magnitude; thresholds increased (degraded) with increasing modulation rate, and thresholds were generally somewhat higher for the SD task than for the FM task. The between-subjects effect of task order was not significant and did not significantly interact with any of the within-subjects effects (*p* > 0.05 in all cases).

The ANOVA also revealed significant interactions between task and rate [*F*(1.94, 35.0) = 23.5, *p* < 0.001, $\eta_{p}^{2}$ = 0.566] and between task and passband [*F*(1, 18) = 12.4, *p* = 0.002, $\eta_{p}^{2}$ =0.407]. Neither the interaction between rate and passband [*F*(3, 54) = 0.777, *p* = 0.512, $\eta_{p}^{2}$ = 0.041] nor the three-way-interaction (task-rate-passband) [*F*(2.36, 42.4) = 1.32, *p* = 0.281, $\eta_{p}^{2}$ = 0.068] was significant.

To further explore the significant interactions, *post hoc* analyses were carried out using Bonferroni corrections for multiple comparisons. An examination of the task-rate interaction with alpha-values Bonferroni-corrected for 4 comparisons (α = 0.0125) revealed that thresholds were higher for the SD than the FM stimuli at the two highest FM rates of 10 and 20 Hz [*t*(18) = −6.01*, p* < 0.001 and *t*(18) = −9.22*, p* < 0.001, respectively] but not the lower FM rates of 2 or 5 Hz [*t*(18) = 0.222*, p* = 0.827 and *t*(18) = −1.86, *p* = 0.08, respectively]. All four comparisons for the task-passband interaction were significant after correcting for multiple comparisons (α = 0.0125): both FM and SD thresholds were lower (better) in the resolved relative to the unresolved passbands [FM: *t*(18) = −35.8, *p* < 0.001; SD: *t*(18) = −34, *p* < 0.001], and FM thresholds were lower than SD in both passbands [resolved: *t*(18) = −5.87, *p* < 0.001; unresolved: *t*(18) = −3.04, *p* = 0.007]. The task-passband interaction primarily reflects the observation that the difference between FM and SD thresholds is greater for stimuli with resolved harmonics than those with unresolved harmonics (SD thresholds greater than FM thresholds by a factor of 1.83 and 1.27 for resolved and unresolved harmonics, respectively).

Overall, the results confirm a large (order of magnitude) difference in thresholds between stimuli with resolved and unresolved harmonics, as expected. However, contrary to our first prediction, the slopes of the function relating FM detection thresholds to modulation rate were similar for the spectrally resolved and unresolved harmonics. The same was true in the SD conditions. A comparison of the FM and SD results also seem to be inconsistent with our second prediction that FM and SD thresholds should be similar and should behave similarly as a function of modulation rate. In contrast, the SD thresholds were higher (worse), and the functions relating thresholds to modulation rate were steeper than in the FM task for both passband conditions.

In summary, FM detection thresholds with spectrally resolved and unresolved harmonics increase (worsen) similarly as a function of modulation rate. Thresholds in the SD conditions worsen more rapidly as a function of rate than thresholds in the FM condition. The difference in thresholds between FM and SD conditions was somewhat greater with resolved than with unresolved harmonics.

## 4. Discussion

**Fig. 2.**
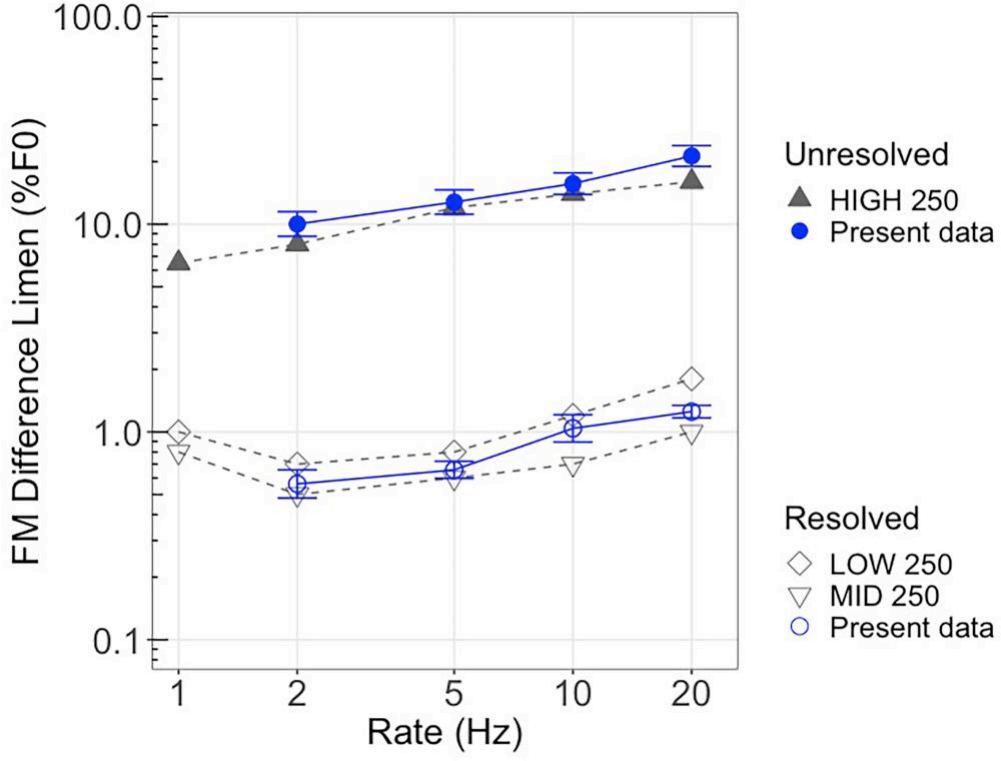
Comparisons of FM detection thresholds across studies. Thresholds are plotted as peak-to-peak frequency change as a function of modulation rate. Error bars for the current data represent ±1 standard error of the mean. Current results for resolved and unresolved passbands of the FM task (solid lines, open and filled blue circles, respectively) are superimposed on the most comparable data from Carlyon *et al.* (2000), who tested six passband conditions across two F0s. Symbols show their results for F0 = 250 Hz complex tones filtered into LOW (125–625 Hz, open gray diamonds), MID (1375–1875 Hz, open gray downward triangles), and HIGH (3900–5400 Hz, filled gray triangles) frequency regions.

Our finding of no interaction between modulation rate and spectral resolvability is not consistent with our first prediction, based on the availability of FM-to-AM cues for resolved but not unresolved harmonics. However, as shown in Fig. 2, our results are in very good agreement with the most comparable conditions studied by Carlyon *et al.* (2000) with a 250 Hz F0 and passbands of 125–625 and 1375–1875 Hz (their LOW and MID regions, respectively) for the spectrally resolved conditions and the passband of 3900–5400 Hz (their HIGH region) for the spectrally unresolved conditions, despite their low number of participants (*N* = 4). Both our and their results show that FM detection thresholds increase with rate similarly for both resolved and unresolved harmonics, in line with our finding of no significant interaction between modulation rate and spectral resolvability.

One way in which our results may be reconciled with an FM-to-AM encoding mechanism is to include an assumption that the processing of out-of-phase AM, as required for detecting FM (Whiteford *et al.*, 2020), may also be sluggish and may exhibit the same rate dependencies as the temporal encoding needed to process FM with only unresolved harmonics. Thus, perhaps both place and time encoding of frequency are limited by the same higher-level constraints. Potential evidence supporting this claim by comparing AM and FM detection is provided by Whiteford and Oxenham (2023).

The finding of no significant difference between FM and SD at slow modulation rates (2 and 5 Hz) is consistent with the results of Plack and Carlyon (1995), who only tested thresholds at 5 Hz and for a single modulation cycle. At that rate, they also reported a somewhat greater difference between FM and SD thresholds for resolved than for unresolved harmonics, also in line with our findings. However, their conclusion that SD and FM thresholds are generally equivalent does not appear to generalize to faster modulation rates of 10 and 20 Hz for either resolved or unresolved harmonics. At face value, this outcome is inconsistent with our second prediction that SD thresholds should reflect FM thresholds in that they both provide similar information, based on the snapshot theory of FM detection. However, the results may be reconciled with the snapshot theory by taking into account higher-level considerations of auditory object formation and the role of stimulus onsets (Demany *et al.*, 2009). Both Plack and White (2000) and Demany *et al.* (2009) have argued that stimulus discontinuities caused by offsets and onsets lead to the perception of multiple objects, which in turn may lead to less efficient between-objects comparisons of pitch than within-object judgments of pitch fluctuations. Plack and White (2000) showed that this “resetting” at offsets and onsets could be overcome by introducing noise that induced the continuity illusion between successive tone bursts (Warren, 1984). In the same vein, Demany *et al.* (2009) found that introducing discontinuities between otherwise continuous tones worsened performance, despite not altering the information available to participants. Although our background noise was sufficient to mask distortion products, its spectrum did not overlap with that of the stimuli, and so it did not mask the discontinuities in the SD stimuli. Thus, our observed differences in thresholds between the SD and FM tasks may reflect the perception of multiple discrete tones, as opposed to a continuously fluctuating tone, and thus may not be inconsistent with the snapshot theory of FM encoding.

In summary, our results do not provide clear support for the idea that FM-to-AM conversion aids in FM detection at fast modulation rates when resolved harmonics are present, as the same effect of modulation rate is observed with unresolved harmonics where AM cues are not available. Also, in contrast to our initial prediction that SD and FM thresholds should remain similar across different modulation rates, we found that in fact SD thresholds worsened more rapidly with modulation rate than did FM detection thresholds. Both findings may be reconciled with existing theories if it is assumed that more central processes, involving across-channel AM detection and auditory object formation, limit performance at fast modulation rates.

## Data Availability

The data that support the findings of this study are available from the corresponding author upon reasonable request.
